# Supplementary material for: The genome sequence of a long-legged fly, Neurigona quadrifasciata (Fabricius, 1781) (Diptera: Dolichopodidae)
Source: Wellcome Open Res. 2025 Oct 24;10:597. [Version 1] doi: 10.12688/wellcomeopenres.24974.1 (PMC12824482; doi:10.12688/wellcomeopenres.24974.1)
Supplement: Peer review history

# Reviewer report: Reviewer response for version 1

**DOI:** 10.21956/wellcomeopenres.27509.r144108

**Published:** 2026-02-03

**Authors:** Kristina Gagalova

**Affiliations:** 1 Curtin University, Perth, Australia

Thank you for submitting the manuscript "The genome sequence of a long-legged fly, *Neurigona quadrifasciata* (Fabricius, 1781) (Diptera: Dolichopodidae)"

 I found the methods and results section clear. I only have a few points for clarification:

 1) Is there any cytological information regarding the number of chromosomes of the species? 

 2) What about the sex chromosomes? Can you please state that clearly in the results?

 3) In genome scope, do you assume that the genome is diploid?

Are sufficient details of methods and materials provided to allow replication by others?

Yes

Is the rationale for creating the dataset(s) clearly described?

Yes

Are the datasets clearly presented in a useable and accessible format?

Yes

Are the protocols appropriate and is the work technically sound?

Yes

Reviewer Expertise:

insect and plant genomics, pipeline development, omics data integration, pangenomics, structural bioinformatics

I confirm that I have read this submission and believe that I have an appropriate level of expertise to confirm that it is of an acceptable scientific standard.

# Reviewer report: Reviewer response for version 1

**DOI:** 10.21956/wellcomeopenres.27509.r144106

**Published:** 2026-01-21

**Authors:** Stuart J.E. Baird

**Affiliations:** 1 Academy of Sciences of the Czech Republic, Národní, Staré Město, Czech Republic

The genome note is a clean example of the DToL pipeline output. I have only one comment: In Figure 4 (Evaluation of k-mer completeness using MerquryFK) there is no evidence of the red curve for primary-only data. If it is obscured behind another curve, this should be explained. It it is absent, this should be explained.

Are sufficient details of methods and materials provided to allow replication by others?

Yes

Is the rationale for creating the dataset(s) clearly described?

Yes

Are the datasets clearly presented in a useable and accessible format?

Yes

Are the protocols appropriate and is the work technically sound?

Yes

Reviewer Expertise:

Admixture genomics. Spatial genetics.

I confirm that I have read this submission and believe that I have an appropriate level of expertise to confirm that it is of an acceptable scientific standard.

# Reviewer report: Reviewer response for version 1

**DOI:** 10.21956/wellcomeopenres.27509.r138603

**Published:** 2025-11-25

**Authors:** Dong Mei Li

**Affiliations:** 1 Ministry for Primary Industries, Auckland, New Zealand

This manuscript presents a chromosomal-level genome sequence for the long-legged fly, *Neurigona quadrifasciata. *In addition, a mitochondrial genome has also been assembled. The manuscript is well-written and provide valuable information for future research, as it delivers a high-quality reference genome for the species – the first reported for the genus *Neurigona*. I do not have major revisions to suggest, but I do have a few comments:

1. **Keywords:**  Most of the listed keywords (e.g. *Neurigona quadrifasciata*; long-legged fly, genome sequences; Diptera) are already in the manuscript. Typically, keywords should not simply repeat words from the title but instead provide additional indexing terms that broaden discoverability. I recommend selecting alternative or complementary keywords (e.g,” “mitochondrial genome,”  “chromosomal assembly or others).
2. **Background:** In the second paragraph, the manuscript provides detailed information on the behavior of males within the genus. However, it would be helpful to also include specific details about the behavior of *N. quadrifasciata* males, rather than only general genus-level traits. In addition, while the last sentence describes male morphology, there is no mention of female morphology. Including information on female characteristics and behavior would provide a more balanced and comprehensive background for readers.
3. **Figure 1:** The current figure would be more informative if the diagnostic features of the fly were clearly highlighted. In addition, the figure legend should indicate the specimen shown is female included the sample ID as well. This would enhance the utility of the figure for readers who may wish to compare diagnostic features across related taxa.
4. **Methods**
  - Several sections of the Methods appear to closely resemble the text from another genome sequence publication in this journal, I suggest to rephrasing these sentences. One example is the second paragraph in the “Sample acquisition and DNA barcoding” section.  
  -  The Qubit Fluorometer is referred to in three different ways: “Qubit Fluorometer,” “Qubit Fluorometer (Thermo Fisher Scientific),” and *“Qubit Fluorometer V4 (Thermo Fisher Scientific).”* For consistency and clarity, the authors should standardize the terminology. It is recommended to use Qubit Fluorometer V4, (Thermo Fisher Scientific) when first introduced, and then refer to it consistently as Qubit Fluorometer throughout the remainder of the manuscript.
  - When Qubit dsDNA High Sensitivity Assay kit first mentioned in the Nucleic acid extraction section, the abbreviation HS should be added immediately after “High Sensitivity”. Thereafter, the kit should be referred to as Qubit HS Assay kit.
5. **Methods – Sample Acquisition and DNA Barcoding**:
  - The manuscript does not provide details on how the two specimens were collected. Please specify the collection methods used (e.g., sweep netting, Malaise trap, hand collection, or other techniques).
  - In the 1 ^st^ paragraph, the manuscript mentions that a second specimen was used for Hi-C sequencing and RNA-Seq. However, unlike the first specimen, the sex of this individual is not specified. Please indicate whether the second specimen was male or female in the Methods section, if relevant, discuss any implications for the results.
  - In the second paragraph of the *“Sample acquisition and DNA barcoding”* section, additional details should be provided for clarity. For example, the phrase “a small sample was dissected from each specimen” is vague; the authors should specify which tissue was used (e.g., leg, thoracic muscle, or abdominal tissue).
  - It is also unclear whether tissue lysis, PCR, and sequencing were performed at the Wellcome Sanger Institute or in the authors’ laboratory. If these steps were not conducted at WSI, please describe the methods used for DNA extraction, PCR amplification, and sequencing.
  - It would be useful to clarify whether identical COI sequences were obtained from both specimens, as this information supports species verification and consistency across samples.
6. **Methods – Nucleic Acid Extraction**
  - In this section, the abbreviation **WSI** should be used instead of spelling out “Wellcome Sanger Institute,” since the abbreviation has already been introduced.
  - More detail is needed regarding the RNA extraction process. Please specify which tissue was used and the approximate amount of tissue (in mg) employed for RNA extraction. Providing these details will improve reproducibility and allow readers to better assess the quality and reliability of the RNA-Seq data.
7. **Methods – PacBio HiFi library preparation bd sequencing **
  - There is an inconsistency in reported DNA fragment size: 9.3 kb is mentioned near the end of page 3, while ~10 kb is noted on page 4. Please ensure consistency throughout the section.
  - The statement “A 0.85x pre-PCR clean-up was carried out with Promega ProNex beads” appears twice in consecutive paragraphs. This repetition should be avoided by consolidating the information into a single clear description.
  - The rationale for dividing the DNA into two aliquots for dual PCR is not explained. Please provide justification for this step, as it will help readers understand the methodological choices made.
8. **Methods – Hi-C:** The manuscript states that 20–50 mg of tissue was used. Since only a single specimen was processed, the exact amount of tissue should be reported rather than a range. In addition, please specify which part of the insect was used for Hi-C sequencing.
9. **Table 1.** It appears that not the whole organism was used for sequencing, since tissue was removed for DNA barcoding.
10. **Assembly statistics:** the manuscript notes that mitochondrial genome was assembled, and its length is mentioned in the Abstract. However, no details are provided in this section about the genome structure or gene annotation. Some details will assist readers to get better understanding of the mitochondrial genome.

Are sufficient details of methods and materials provided to allow replication by others?

Partly

Is the rationale for creating the dataset(s) clearly described?

Yes

Are the datasets clearly presented in a useable and accessible format?

Yes

Are the protocols appropriate and is the work technically sound?

Yes

Reviewer Expertise:

Molecular biology and DNA/RNA sequencing methods, genomics, bioinformatics, Molecular Diagnostics, Mitochondrial genome, Phylogeny, Real-time PCR assay development.

I confirm that I have read this submission and believe that I have an appropriate level of expertise to confirm that it is of an acceptable scientific standard.
